# Diversity of Murine Norovirus Strains Isolated from Asymptomatic Mice of Different Genetic Backgrounds within a Single U.S. Research Institute

**DOI:** 10.1371/journal.pone.0021435

**Published:** 2011-06-28

**Authors:** Elyssa L. Barron, Stanislav V. Sosnovtsev, Karin Bok, Victor Prikhodko, Carlos Sandoval-Jaime, Crystal R. Rhodes, Kim Hasenkrug, Aaron B. Carmody, Jerrold M. Ward, Kathy Perdue, Kim Y. Green

**Affiliations:** 1 Laboratory of Infectious Diseases, National Institute of Allergy and Infectious Diseases, National Institutes of Health, Department of Health and Human Services, Bethesda, Maryland, United States of America; 2 Retrovirus Immunology Section, National Institute of Allergy and Infectious Diseases, National Institutes of Health, Department of Health and Human Services, Hamilton, Montana, United States of America; 3 Comparative Medicine Branch, National Institute of Allergy and Infectious Diseases, National Institutes of Health, Department of Health and Human Services, Bethesda, Maryland, United States of America; Tulane University, United States of America

## Abstract

Antibody prevalence studies in laboratory mice indicate that murine norovirus (MNV) infections are common, but the natural history of these viruses has not been fully established. This study examined the extent of genetic diversity of murine noroviruses isolated from healthy laboratory mice housed in multiple animal facilities within a single, large research institute- the National Institute of Allergy and Infectious Diseases of the National Institutes of Health (NIAID-NIH) in Bethesda, Maryland, U.S. Ten distinct murine norovirus strains were isolated from various tissues and feces of asymptomatic wild type sentinel mice as well as asymptomatic immunodeficient (RAG 2^−/−^) mice. The NIH MNV isolates showed little cytopathic effect in permissive RAW264.7 cells in early passages, but all isolates examined could be adapted to efficient growth in cell culture by serial passage. The viruses, although closely related in genome sequence, were distinguishable from each other according to facility location, likely due to the introduction of new viruses into each facility from separate sources or vendors at different times. Our study indicates that the murine noroviruses are widespread in these animal facilities, despite rigorous guidelines for animal care and maintenance.

## Introduction

Noroviruses, members of the virus family *Caliciviridae*, are ubiquitous enteric viruses found in a broad range of animal species, including mice [1]. In humans, they are associated with nausea, vomiting, diarrhea, and fever that characteristically last approximately 24–48 hours. They are transmitted via person-to-person contact, or by exposure to contaminated food or water. Outbreaks often occur in settings such as nursing homes and cruise ships, where close contact allows for easy spread of the virus. Long-term illness and shedding of noroviruses have been reported in immunocompromised individuals [2]. The approximately 7.6 kb positive-sense, single-stranded RNA genome of norovirus is organized into three major open-reading frames (ORFs): ORF1 encodes a large polyprotein that is cleaved into nonstructural precursors and proteins involved in viral replication; ORF2 encodes the major capsid protein, VP1; and ORF3 encodes a minor structural protein of the virion, VP2 [3], [4], [5], [6]. In murine norovirus (MNV) strains [7], an additional ORF4 has been described that encodes a protein of unknown function [8]. Noroviruses have been divided into five distinct genogroups (GI–GV) [9], with human pathogens belonging to GI, GII, and GIV. The bovine and murine noroviruses detected thus far belong to GIII and GV, respectively. Although genetically distinct from the human noroviruses, the murine noroviruses have emerged as an important model in the study of norovirus biology and replication because, in contrast to the human pathogens, an efficient cell culture system is available [10], [11].

The discovery of the first MNV strain, designated MNV-1, in mice within a U.S. university research facility [7] led to the development of antibody detection assays that quickly showed serologic evidence for a widespread distribution of this virus in laboratory mice [12]. Although most mice infected with MNV appear healthy, the viruses have been associated with severe systemic illness and death in certain genetically engineered mice lacking an intact innate immune system such as STAT1^−/−^ mice [7]. The MNV strains characterized thus far have formed a highly related single genotype within GV, whether associated with asymptomatic infection or disease [8], but recombination events between viruses have been proposed [13]. At our research institute, the National Institute of Allergy and Infectious Diseases (NIAID) of the National Institutes of Health (NIH), U.S., serologic evidence for MNV infection was found in several “sentinel” mice in various animal rooms [14], [15], which prompted us to examine the molecular epidemiology of MNV within our animal facilities. We isolated murine noroviruses in cell culture from ten NIH mice and the genomes of these viruses were sequenced. The MNV strains characterized within each NIAID animal facility were distinct from those in other facilities of the institute, suggesting the introduction of the virus into each facility from a separate source. However, the NIH MNV strains showed marked overall genetic relatedness with other murine norovirus strains detected thus far.

## Results

**Figure 1:**
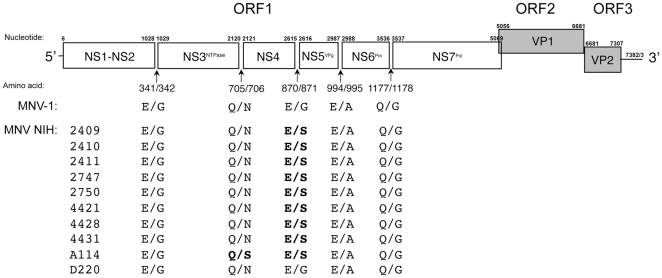
Sequence analysis of NIH MNV strains. Genome organization of prototype strain MNV-1 [7] and ORF1 polyprotein cleavage sites [6] in comparison with the predicted cleavage sites in the ORF1 polyprotein of the NIH murine norovirus strains. Dipeptide recognition sites that differ from MNV-1 are indicated in bold type.

By serologic screening of sentinel mice housed in several NIAID animal facilities, we found evidence initially for norovirus infection, and in addition, we were able to detect the virus by RT-PCR in representative mice [15]. This study focused on the further characterization of MNV strains isolated from mice housed in three of the animal facilities (Table 1). While all facilities housed mice from the same commercial vendors, each facility also housed mice received from separate non-commercial sources. Ten MNV strains (designated as the NIH strains) were isolated following passage in RAW264.7 cells, and their full-length genomes were sequenced. The genomes were similar in length (7382 or 7383 nt) and organized into three major open reading frames (ORFs): ORF1 (nt 6–5069) encoding the nonstructural polyprotein, ORF2 (nt 5056–6681) encoding the major capsid protein, VP1, and ORF3 (nt 6681–7307) encoding the minor capsid protein, VP2 (Figure 1). In addition, the genomes contained a predicted conserved fourth ORF (ORF4) spanning nt 5069–5710 that would encode a putative protein of 23.6 kDa [8].

**Table 1:** Murine norovirus strains isolated from NIH mice.

Strain Designation	NIH Facility	Mouse	Specimen	Passage (P)
		Strain	Source for Virus	Number*
Mu/NoV/GV/NIH- 2409/2005/US	B14	RAG2−/−	MLN	P0, P1, P2, 3xPP
Mu/NoV/GV/NIH -2410/2005/US	B14	RAG2−/−	MLN	P3
Mu/NoV/GV/NIH- 2411/2005/US	B14	RAG2−/−	MLN	P3
Mu/NoV/GV/NIH- 2747/2005/US	B14	RAG2−/−	MLN	P3
Mu/NoV/GV/NIH- 2750/2005/US	B14	RAG2−/−	MLN	P3
Mu/NoV/GV/NIH -4421/2005/US	B14	RAG2−/−	Duodenum	P3
Mu/NoV/GV/NIH -4428/2005/US	B14	RAG2−/−	Duodenum	P3
Mu/NoV/GV/NIH-4431/2005/US	B14	RAG2−/−	Duodenum	P4
Mu/NoV/GV/NIH -A114/2006/US	TB	Swiss Webster	Fecal	P7
Mu/NoV/GV/NIH-D220/2007/US	B50	Swiss Webster	Fecal	P2

*Passage (P) number of virus in RAW264.7 cells at which genome sequence was determined as described in text.

Comparison of the nucleotide sequences showed a close genetic relationship among the NIH MNV strains, with a range of 87.2–99.7% overall nucleotide identity. The nucleotide identities with the prototype strain, MNV-1, ranged from 87.4–89.1%, and with all other full-length MNV sequences available in GenBank, the identities ranged from 87.4–99%. Comparison of the deduced amino acid sequences among the NIH strains showed that the proteinase was the most highly conserved protein (approximately 97% similarity), while the VP1 (major capsid protein) showed the most variability (as low as 89.6% similarity). Analysis of the MNV-1 VP1 capsid protein showed that the shell (S) region of the capsid protein (amino acid residues 49 to 218) was 100% conserved among the NIH MNV strains while the protruding (P) region (amino acids 229 to 537) was most variable, consistent with data reported for other highly-related murine noroviruses [8]. The P2 domain (amino acids 278–415) within the P region is exposed predominantly on the surface of the virion and has been proposed as the major site of immune pressure and receptor binding for calicivirus virions [16]. Consistent with this, passage of the virulent MNV-1 strain in cell culture yielded virus in which lysine was changed to glutamic acid at position 296 in the P2 domain. This single amino acid substitution resulted in loss of virulence of the MNV-1 strain in STAT1-deficient mice (17). Of interest, a glutamic acid residue at position 296 was conserved in the cell-culture adapted NIH MNV strains in this study, as well as the NIH-2409 virus prior to adaptation (data not shown) [17]. A leucine residue at position 386 involved in the formation of a neutralization epitope in MNV-1 [18] was conserved in the P2 domain of the NIH MNV strains (data not shown).

The replication strategy for murine norovirus involves the proteolytic processing of the ORF1 polyprotein into several nonstructural proteins by the virus-encoded cysteine proteinase (Pro). The proteinase mediates five cleavages at dipeptide sites ^341^E/G^342^, ^705^Q/N^706^, ^870^E/G^871^, ^994^E/A^995^, and ^1177^Q/G^1178^ to produce six proteins: NS1-2 (Nterm), NS3 (NTPase), NS4 (p18), NS5 (VPg), NS6 (Pro) and NS7 (Pol) [6]. Alignment of the deduced ORF1 polyprotein sequences of the NIH MNV strains with that of MNV-1 showed that these dipeptide cleavage sites were conserved in location, but with some minor variation in the P1′ positions at amino acid residues 706 and 871 (Figure 1).

**Figure 2:**
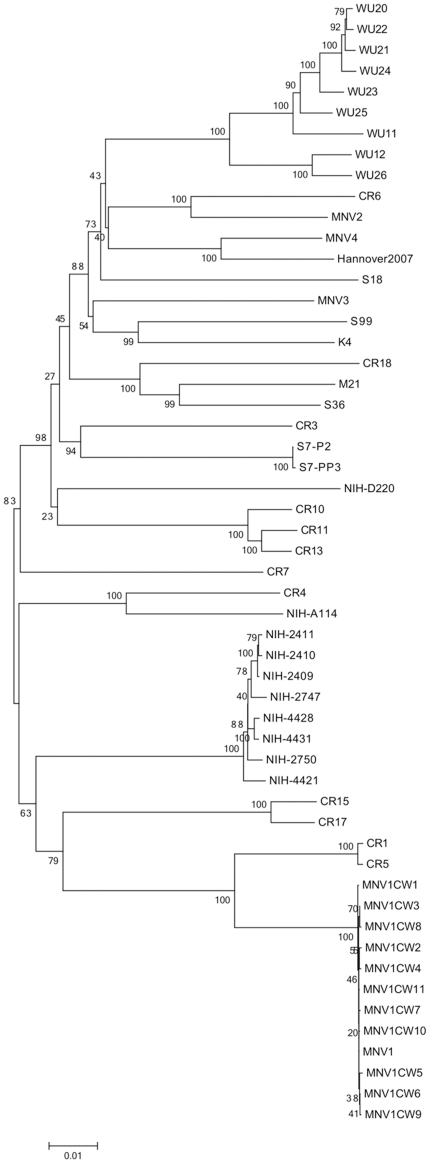
Phylogenetic analysis of NIH MNV viral genomes in comparison with representative viruses previously published. Phylogenetic relationships were inferred using Neighbor-joining method, with the evolutionary distances computed by the Tamura-Nei method. The statistical support for tree nodes was evaluated by bootstrap analysis (500 replicates) and values higher than 65 are shown above the corresponding branches. The strains in this analysis and their GenBank accession numbers are shown in the Supplemental Table (Table S1).

A phylogenetic analysis of the complete genome sequences of the NIH MNV strains was performed to compare their relatedness with that of other murine noroviruses (Figure 2). The NIH strains clustered with Genogroup V murine noroviruses, but did not share complete identity with other strains reported thus far. Eight of the strains formed a distinct monophyletic group (NIH- 4421, 2747, 2409, 2411, 2410, 2750, 4428, and 4431, with nucleotide variation not exceeding 1%). Strain NIH-A114 did not cluster with other viruses and formed a distinct branch. Strain NIH-D220 clustered most closely with a phylogenetic group that included several Charles River (CR) and Washington University (WU) strains. The three genetic groupings of the NIH strains corresponded to their isolation from three distinct animal facilities in the institute at different times (Table 1).

**Figure 3:**
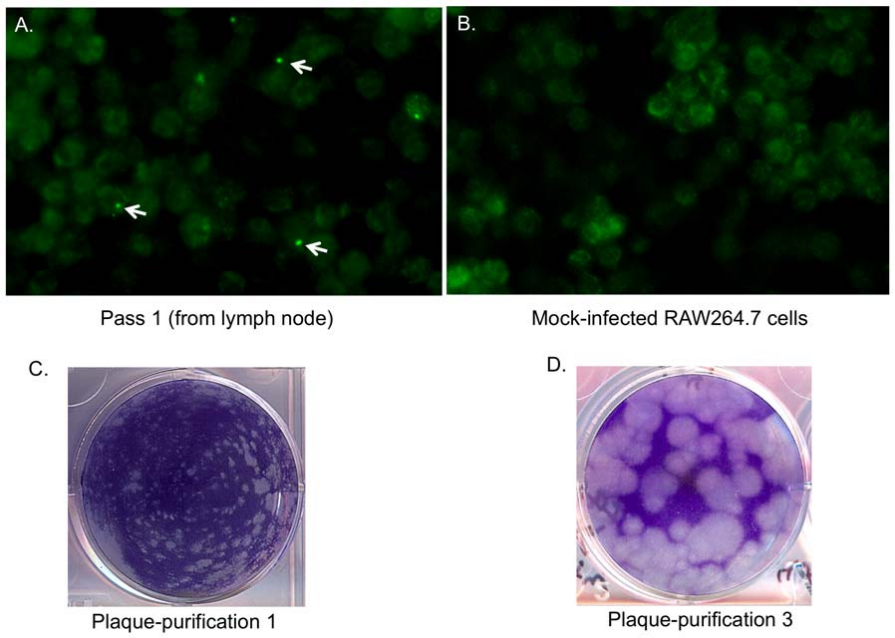
Serial passage of MNV NIH-2409 in RAW264.7 cells. **A.** Immunofluorescence assay of RAW264.7 cells inoculated directly with a lymph node homogenate from mouse. Medium was removed after 7 days, cells were fixed with methanol, and viral antigen expression was detected with anti-MNV-1 VP1 serum. Representative positive MNV antigen expression is indicated by white arrows. **B.** Mock-infected RAW264.7 cells incubated with same serum. **C.** First plaque purification of NIH MNV-2409 following P3 in RAW264.7 cells as described in Material and Methods. **D.** Plaque morphology after two additional plaque purifications in RAW264.7 cells (3XPP virus stock).

A previous study had identified amino acid substitutions in the NS4 and VP1 proteins that emerged during cell-culture passage of the MNV-1 strain. In this study, we followed the evolution of MNV NIH-2409 during cell culture passage to examine whether similar substitutions occurred. Strain NIH-2409 was isolated from a RAG2^−/−^ mouse in a facility (B14) that has operated for more than 20 years, and that accepts vendor animals and transfer animals from within NIH as well as imported animals. The mesenteric lymph node tissue was homogenized and added to a monolayer of RAW264.7 cells and incubated for 7 days. There was no visible cytopathic effect (CPE) in RAW264.7 cells following the initial 7-day infection period. However, an immunofluorescence assay showed the presence of occasional cells with a perinuclear, localized area of positive reactivity with the MNV-1-specific antibodies (as indicated by arrows in Figure 3A) that was not present in the mock-infected RAW264.7 cells (Figure 3B). The passage 1 (P1) material was added to a new monolayer and the visible CPE increased with each subsequent cell passage. After three passages, the material was subjected to plaque purification, and small plaques were observed (Figure 3C). Following two additional rounds of plaque purification (selecting large plaques), the plaque morphology resembled MNV-1 (Figure 3D).

The “consensus sequence” of the genome of the virus in the original MLN specimen (P0) was determined by the direct sequencing of RT-PCR products that spanned the genome. Only a few nucleotide changes were observed between the first two serial passages (P1 and P2) and after three rounds of plaque purification (3xPP), consistent with previous studies of MNV-1 [11] (Table 2). At nucleotide position 2166, a mixed population was detected that would result in either a serine or alanine at amino acid residue 721 in the NS4 region of the ORF1 polyprotein. Following three rounds of plaque purification of P3, an alanine became the predominant sequence at position 721. Of note, no adaptive amino acid mutations were observed in the VP1 (capsid) protein for this strain.

**Table 2:** Sequence analysis of viral genomes during passage of MNV NIH-2409 in cell culture.

Open Reading Frame (ORF)	Protein	Genomic Position of Nucleotide	Position of Amino Acid	Amino Acid	2409 Lymph Node (Pass 0)	2409 Pass 1	2409 Pass 2	2409 Pass 3 after plaque purification (3xPP)
ORF1	NS1-2	188	61	Ala	GCA	GCA /GCG	GCG /GCA	GCA
	NS1-2	320	105	Ala	GCC	GCT	GCT	GCC
	NS1-2	629	208	Pro	CCC	CCT	CCT	CCC
	NS3	1469	488	Arg	CGT/CGC	CGC/CGT	CGC/CGT	CGC
	NS3	1487	494	Arg	CGG /CGA	CGA /CGG	CGA /CGG	CGA
	NS3	1817	604	Thr	ACT /ACC	ACC /ACT	ACC /ACT	ACC
	NS3	2081	692	Val	GTC /GTT	GTT /GTC	GTC /GTT	GTC
	NS4	2166	721	Ser→Ala	TCC/ GCC	GCC/ TCC	TCC/ GCC	GCC
	NS4	2495	830	Tyr	TAC	TAT /TAC	TAC /TAT	TAT
	NS4	2531	842	Tyr	TAC	TAT /TAC	TAC /TAT	TAC
	NS4	2543	846	Gly	GGT /GGC	GGT /GGC	GGT /GGC	GGT
	NS7	3803	1266	Val	GTT /GTC	GTC /GTT	GTC /GTG	GTC
	NS7	3809	1268	Asp	GAT /GAC	GAT /GAC	GAT /GAC	GAC
	NS7	4151	1382	Phe	TTC /TTT	TTC /TTT	TTT /TTC	TTC
ORF2	VP1	5481	142	Thr	ACA	ACG /ACA	ACA /ACG	ACG
	VP1	5892	279	Thr	ACG /ACC	ACG	ACG	ACG
	VP1	5904	283	Gly	GGC /GGT	GGC	GGC	GGC
	VP1	6550	499	Leu	TTG	CTG/ TTG	TTG/ CTG	CTG

Note: Positions with variation in sequence are underlined.

## Discussion

Murine noroviruses are associated with asymptomatic infection and shedding in many mouse strains (both normal and genetically-modified), making the viruses difficult to detect in animals without active screening [13]. We investigated the characteristics of several murine norovirus strains within our research institute (NIAID) in order to gain insight into norovirus diversity and natural history within a single setting. A real-time qRT-PCR assay [19] that readily detects MNV strains in mice housed in the NIAID animal facilities in Hamilton, Montana (data not shown) proved efficient in the detection of MNV strains in Maryland. Ten unique MNV strains (designated MNV NIH) were isolated from tissue and stool samples collected from asymptomatic laboratory mice that were housed in rooms with MNV-positive sentinel mice. The ten NIH murine norovirus strains clustered within Genogroup V, similar to all MNV strains characterized thus far. However, the strains fell into three distinct lineages within Genogroup V. Viruses isolated from mice within the same NIAID facility were distinct compared with those from mice in other facilities on campus. This observation suggests that strains are introduced independently from different sources as they are brought into each animal facility of the institute.

One MNV strain (NIH-2409) was selected for additional analyses in order to identify mutations in the genome involved in cell culture adaptation. Unlike MNV-1, which was highly cytopathic upon its initial isolation in cell culture [10], strain MNV NIH-2409 did not cause visible CPE in early passages. Interestingly, a single amino acid change was detected in the NS4 (“3A-like”) protein following adaptation. Serine was the predominant amino acid at residue 721 in viral RNA purified from the original lymph node tissue, but alanine was the predominant amino acid residue after passage and plaque purification. This variation was similar in location to an amino acid residue (position 716) that changed from valine to isoleucine in NS4 of the MNV-1 strain following passage in RAW264.7 cells [10]. The function of the NS4 protein has not been determined, but it is likely that this protein may play a role in interactions with host cells. The majority of the synonymous (silent) nucleotide mutations during cell culture passage occurred in ORF1 and ORF2 in both NIH-2409 and MNV-1. Strain NIH-2409 showed no changes in ORF3 during serial passage, while MNV-1 demonstrated one silent change (nt 6770) during passage and plaque purification.

It is not clear when MNV was introduced into laboratory mice, and whether such strains circulate in wild mice. Recent studies of experimental mouse models found that the presence of MNV did not affect the immune response and recovery from Friend retrovirus [19], or the development of adaptive immunity to vaccinia virus and influenza virus [20]. Murine norovirus did not affect experimental outcomes from murine cytomegalovirus infection, although there was a decreased CD8 T cell response [21]. However, the presence of MNV did increased the length of time have an effect on parvovirus was shedding in Balb/c mice [22] and concerns have been raised for its effect in other murine disease models [23], [24], [25]. Sequence analysis of the new MNV NIH strains described in this study contributes to the growing database of genomic norovirus sequences. Continued analysis of the molecular epidemiology of these viruses should give insight into their natural history and evolution in mice.

## Materials and Methods

### Cells and viruses

The murine macrophage-like cell line RAW264.7 was obtained from American Type Culture Collection (Manassas, VA). The cells were grown in Dulbecco's Modified Eagle's Medium (DMEM) supplemented with penicillin (250 units/ml), streptomycin (250 µg/ml), 1% glutamine, and 10% heat-inactivated fetal calf serum. Murine norovirus strain MNV-1 [7] was a gift of Dr. Herbert Virgin, Washington University, Saint Louis, MO. Mesenteric lymph node (MLN), hepatic, spleen or duodenal tissues and fecal samples were collected from mice housed in three different NIAID animal facilities on the NIH, Bethesda, Maryland campus [15] (Table 1) under a protocol approved by the NIAID Animal Care and Use Committee. Sentinel mice in these facilities were routinely monitored for serological evidence of infection with several agents, including MNV, and all three facilities reported MNV antibody-positive sentinel mice. Fifty µl of the filtered tissue or fecal suspension was added to a RAW264.7 cell monolayer seeded in one well of a 6-well tissue culture plate (Costar, Corning Inc., Corning, NY), after which the cells were incubated for 5–7 days at 37°C. Cells were then frozen and thawed and 50 µl of the resultant suspension was used to infect a new RAW264.7 cell monolayer. The material was passed between 2 and 7 times, depending on the amount of associated CPE. Fluid from the final passage was collected and subjected to three rounds of plaque-purification. A plaque from the third purification was isolated and amplified to serve as a virus stock for sequence analysis of the complete genome.

### Tissue culture adaptation of MNV strain NIH-2409

An MLN homogenate from mouse 2409 was added to RAW264.7 cells in two wells of a 6-well plate. The material from one well was passed three times in RAW264.7 cells as described above. The second well was fixed with methanol and the presence of MNV antigen after 7 days was examined by immunofluorescence assay as described below. Following pass 3 (P3), the isolated MNV strain, designated NIH-2409, was plaque-purified three times (3XPP). Briefly, a plaque was removed from the monolayer with a pipette tip, diluted in medium, and then subjected directly to a second plaque-purification. A second plaque was removed, diluted in medium, and subjected directly to a third plaque purification. The final isolated plaque was amplified in cells and the virus stock was designated 3XPP. The consensus sequence of the genome of the virus present in the original tissue (P0) was determined and compared to that of the virus obtained at P1, P2, and after three rounds of plaque purification (3XPP).

### RNA extraction

Tissue samples were placed in 0.5 ml sterile, cold phosphate buffered saline (PBS) and disrupted on ice with either a Dounce homogenizer or an electric high shear tissue homogenizer (Omni International, Kennesaw, GA). Tissue suspensions were then stored at −70°C. Fecal pellets were added to PBS at a concentration of approximately 10% w/v in a sterile microfuge tube, followed by vortexing. The solid material in the feces was pelleted by low-speed centrifugation (10,000 rpm×10 minutes) in a tabletop microfuge and the clarified fecal suspension was filtered and transferred to a sterile microfuge tube. Viral RNA was extracted with the QIAamp Viral RNA mini kit (Qiagen, Valencia, CA) using a 100 µl aliquot of either the fecal or tissue suspension. The extracted RNA was eluted in 50 µl of RNase-free water and stored at −70C°.

### Sequence analysis of RT-PCR products

The purified RNA was amplified by RT-PCR with the One Step RT-PCR kit (Invitrogen, Carlsbad, CA) in a strategy that produced seven amplicons that were gel-purified and sequenced directly (primers for RT-PCR shown in Table 3). Sequencing was performed with the BigDye Terminator v.3.1 cycle Sequencing Ready Reaction kit in an AB1 3100 Automated sequencer (Applied Biosystems, Foster City, CA). The precise ends of the genome were determined with the 5′ and 3′ Rapid Amplification of cDNA Ends (RACE) systems from Invitrogen.

**Table 3:** Primer pairs used in the amplification of NIH murine norovirus genomes.

**Region 1**	
MNV-1 BsmBl/T7pro/5′-endF	ATATATATATCGTCTCAAAATTCGTCTCACTGGTAATACGACTCACTATAGTGAAATGAGGATGGCAACGCCATCTTCTGCGCCCTCTGTGCGC
MNV-1 n1139R	GCAAATTTGCCCATCTCTTGGGCGGCCTTCAGGGCC
**Region 2**	
NIH MNV-1n922F	GCATAGTCAATGCCCTGATTCTTCTTGCTGAGC
NIH MNV-1n2615R (VPg)	CCCCCGGCCTCTCTTGTTCTTGCCCTTCTTGCTC
**Region 3**	
NIH MNV-1n2392F	GTGTCAGGAGGATCAAAGAGGCTCGCCTTCGCTGC
NIH MNV-1n3734R	CGCGTGGCTCTGAATAGGGCTTGAGCTGGTCTCGC
**Region 4**	
NIH MNV-1n3394F	CAGGTGACTGTGGCTGCCCCTATGTCTACAAGAAGGGAAAC
NIH MNV-1n4840R	GCAGGGCCATCAATTGGGAGGGTCTCTGAGCGTGTC
**Region 5**	
NIH MNV-1n4450F	GCCCCTGCACCACGCAGCTGAACAGCTTGG
NIH MNV-1n5722R	CACGGGCAAGTCGACCATCCGGTAGATGGTTCTCTC
**Region 6**	
NIH MNV-1 n5332F	GCCCACCTCTCAGCCATGTACACCGGCTGGGTTGGGAAC
NIH MNV-1n6733R	CGCCTGAAGGTTTTGGACATTTGAGATGGAATTGC
**Region 7**	
NIH-MNV n6427F	GTCTCCTGGTTCGCGTCTAACGCGTTCACCGTGCAGTCC
NIH-MNV 3′/polyA R	TTTAAAATGCATCTAAATACTACCAAAAGAAAAGAG

### Phylogenetic analysis

Multiple sequence alignments were generated with the ClustalW algorithm in the MacVector, Inc. (Cary, NC) software package. Phylogenetic relationships were inferred using the Neighbor-joining method [26], with the evolutionary distances computed by the Tamura-Nei method [27]. The statistical support for tree nodes was evaluated by bootstrap analysis (500 replicates) [28]. Evolutionary analyses were conducted with the MEGA software package [29].

### qRT-PCR detection assay

A real-time qRT-PCR assay was adapted for the detection of MNV RNA [19], [30]. Briefly, a forward primer 5′-AATCTATGCGCCTGGTTTGC-3′ and a reverse primer 5′-CTCATCACCCGGGCTGTT-3′ were employed to amplify a region of the genome corresponding to nucleotides (nt) 5585–5646. A Taqman probe with the sequence 5′-CACGCCACTCCGC-3′ (corresponding to nt 5613–5625 of the MNV NIH-2409 genome) was generated with FAM dye linked to the 5′-end and BHQ dye at the 3′-end. Five µl of RNA were added to an RT-PCR mix (Brilliant II qRT-PCR core reagent kit, Stratagene, La Jolla, CA). The reaction was then incubated at 45°C for 30 min, 95°C for 10 min, followed by 45 cycles at 95°C for 30 sec, 50°C for 1 min and 72°C for 30 sec in an ABI 7900 HT instrument (Applied Biosystems). Each sample was tested in duplicate and an appropriate triplicate standard curve for absolute quantification was included in each run.

### Immunofluorescence

RAW264.7 cells were infected with virus and incubated 7 days before fixation with cold methanol (100%). The methanol was removed and the cells were rinsed with PBS, pH 7.4. Hyperimmune serum raised in a guinea pig against recombinant MNV-1 VP1 [14] was added at a 1∶100 dilution and incubated at 37°C for one hour. The wells were then rinsed three times with PBS, and affinity purified goat antibodies specific for guinea pig immunoglobulin G (IgG) (ICN Biomedicals, Aurora, OH) and conjugated to fluorescein isothiocyanate were added at a 1∶20 dilution to detect binding of the primary antibody.

## Supporting Information

Table S1 Murine norovirus strains included in phylogenetic analysis and their GenBank accession numbers are shown.
(XLSX)
